# *TP53 *mutation p.R337H in gastric cancer tissues of a 12-year-old male child - evidence for chimerism involving a common mutant founder haplotype: case report

**DOI:** 10.1186/1471-2407-11-449

**Published:** 2011-10-17

**Authors:** Edaise M da Silva, Maria Isabel W Achatz, Ghyslaine Martel-Planche, André L Montagnini, Magali Olivier, Patricia A Prolla, Pierre Hainaut, Fernando A Soares

**Affiliations:** 1 Hospital AC Camargo, Fundação Antonio Prudente, São Paulo, Brazil; 2 International Agency for Research on Cancer, 150 Cours Albert Thomas, 69372 Lyon, France; 3 Department of Gastroenterology, School of Medicine, São Paulo University, São Paulo, Brazil; 4 Service of Medical Genetics, Hospital de Clínicas de Porto Alegre and Department of Genetics, Federal University of Rio Grande do Sul, Porto Alegre, Brazil

## Abstract

**Background:**

Gastric adenocarcinoma is rare in children and adolescents, with about 17 cases under age 21 in the world's literature. We report a case of invasive well-differentiated metastatic gastric cancer in a Brazilian 12-year-old boy without documented familial history of cancer.

**Case presentation:**

The patient, diagnosed with metastatic disease, died seven months after surgery. DNA from intra-surgical specimens revealed a *TP53 *mutation at codon 337 (p.R337H) in samples with neoplastic cells (dysplasia, tumor and metastasis) but not in non-transformed cells (incomplete intestinal metaplasia and non-involved celiac lymph node). In all mutation-positive tissues, p.R337H occurred on the same background, a founder allele identified by a specific haplotype previously described in Brazilian Li-Fraumeni syndrome patients. The same mutant haplotype, corresponding to a founder mutation present in 0.3% of the general population in Southern Brazil, was found in the genome of the father. Presence of this inherited haplotype in the tumor as well as in the father's germline, suggests a rare case of microchimerism in this patient, who may have harbored a small number of mutant cells originating in another individual, perhaps a dizygotic twin that died early in gestation.

**Conclusion:**

This case represents one of the earliest ages at diagnosis of gastric cancer ever reported. It shows that cancer inheritance can occur in the absence of an obvious germline mutation, calling for caution in assessing early cancers in populations with common founder mutations such as p.R337H in Southern Brazil.

## Background

Gastric cancer is the 4^th ^most common cancer in both genders in Brazil, as observed in population-based cancer registries across the country, with incidence rates varying from 22.4 to 53.6 per 100.000 individuals in the States of Goiania and São Paulo, respectively [1]. Despite recent reduction in incidence and mortality observed in the country, occurrence of gastric cancer remains amongst the highest worldwide, in contrast with the sharp decline observed in many countries over recent years [2,3]. The reasons for high incidence are still unclear and are likely to involve multiple etiological factors.

In recent studies on cancer occurrence in Li-Fraumeni syndrome subjects who are carriers of germline *TP53 *mutations in Brazil, gastric cancer appears to be relatively common, representing the 4^th ^most common type of cancer [4]. Germline *TP53 *mutations are the underlying genetic defect of Li Fraumeni Syndrome (LFS) and Li-Fraumeni-like Syndrome (LFL), a set of familial syndromes of predisposition to multiple, early cancer, characterized by an excess risk of adrenocortical carcinoma in early childhood, soft tissue sarcoma, bone sarcoma and brain tumors in adolescence, breast cancer in young adult women and multiple other cancers later in life [5]. An excess risk of colorectal cancer in young adults has been reported as well as earlier occurrence of several common cancers as compared to the general population [6,7].

In Southern Brazil, occurrence of LFS/LF appears to be frequently associated with a *TP53 *mutation at codon 337 (c.1010G > A, Arg337His). The mutation is borne by a specific *TP53 *haplotype characterized by the allele T of SNP rs9894946, located in the 3' non-coding region of the *TP53 *gene. Data from two states in Brazil (Parana and Rio Grande do Sul) indicate that this mutant haplotype is present in about 0.3% of the general population, which is much higher than the estimated frequency of other *TP53 *germline mutations [8]. This mutation, which affects a residue of the oligomerization domain of the p53 protein, has particular functional and regulatory properties that differ from most common LFS *TP53 *mutations. Structural studies have shown that the mutation affects the capacity of p53 to form oligomers with high DNA-binding capacity in a pH dependent manner [9]. This dependence upon pH may explain the incomplete penetrance and the wide phenotypic variations observed in many families carrying the p.R337H mutation.

In this report, we describe a case of gastric cancer in a 12 year-old-boy with no documented familial history of cancer. Given the high frequency of the p.R337H mutation in the Brazilian population, we have analyzed surgical biopsies of this patient for the presence of this particular mutation. The mutation was found in biopsies containing tumor, but not in those with non-neoplastic tissues. The father was also found to carry the same germline p.R337H *TP53 *mutation. This observation highlights one of earliest cases of gastric cancer ever described and identifies the *TP53 *mutant p.R337H as a possible underlying genetic cause.

## Case Presentation

A 12-year-old boy with no documented familial history of cancer presented weakness, malaise, abdominal pain, dysphagia and loss of 14 kg before referral. Upper digestive endoscopy detected a 3.5 cm-ulcerated lesion in the cardia. Surgical exploration revealed an inoperable gastric tumor with adjacent lymph-node involvement, histopathologically proven liver metastases and probable diaphragm, esophageal and celiac axis infiltration. A gastrostomy was performed and the patient received chemotherapy without significant response. The patient died with disseminated disease after seven months.

### Methods

The patient was referred to the Department of Abdominal Surgery of Hospital AC Camargo, São Paulo, Brazil, and biopsies were obtained during diagnostic endoscopy and surgery. The biopsied samples included tissues fixed in 10% buffered formalin and embedded in paraffin (FFPE, Formalin-Fixed, Paraffin-Embedded) and a frozen liver metastasis sample. DNA was extracted from areas of gastric tissue sections defined by a pathologist as normal, metaplastic, dysplatic or cancerous. Blood from relatives was obtained after genetic counseling and informed consent. DNA was extracted from FFPE tissues and white blood cells using standard protocols. *TP53 *mutations were detected by direct sequencing of the entire coding region of the gene using an automated procedure and protocols as described elsewhere [10]. Immunohistochemistry was performed using the following antibodies for p53 (DO-7, DakoCytomation, Glostrup, Denmark), E-cadherin (36, BD Transduction, San Jose, CA), MLH1 (G168-728, BD Biosciences Pharmigen, San Jose, CA), MSH2 (G219-1129, BD Biosciences Pharmigen, San Jose, CA), PMS2 (A16-4, BD Biosciences Pharmigen, San Jose, CA) and MSH6 (44/MSH6, BD Biosciences Pharmigen, San Jose, CA) expression.

### Genetic and Molecular Pathology findings

**Figure 1:**
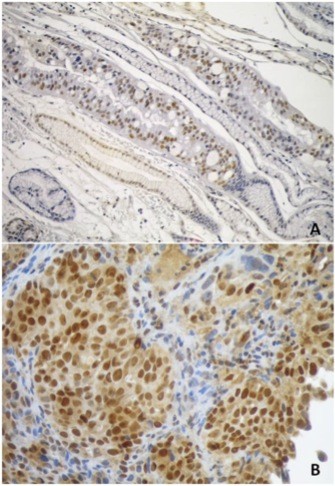
**Immunodetection of p53 in endoscopic (A) or surgical (B) biopsies**. A) endoscopic biopsy in the cardia region, showing areas of metaplasia including areas with intestinal type. Sections were immunostained with anti-p53 DO7 (Dako) using standard protocols. Stromal tissue was negative. Bar: 10 μm;

**Figure 2:**
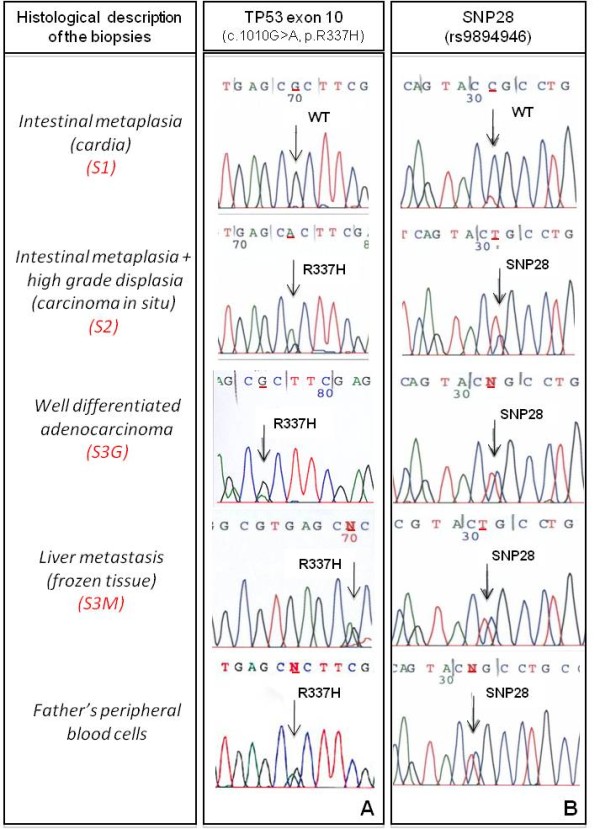
**Sequence at codon 337 of *TP53 *and at SNP 28 (rs9894946) in DNA extracted from patient's biopsies (S1, S2, S3G and S3M) and from the father's peripheral blood cells**. Direct sequencing was performed as described in (http://www-p53.iarc.fr/Download/TP53_DirectSequencing_IARC.pdf) A) As indicated by arrows, metaplasia (S1) showed the wild-type sequence of codon 337 (G allele in homozygosis). The dysplastic (S2), the neoplastic (S3G) and the metastastic (S3M) tissues were heterozygous at codon 337, with a G to A transition corresponding to the p.R337H mutation. B) Sequencing for SNP28 showed the wild-type sequence (CCG) in S1 and the presence of T allele in S2, S3G and S3M. The mutant haplotype was detected in father's germline.

Endoscopic biopsies included intestinal metaplasia in the cardia region (S1) and high-grade dysplasia/in situ gastric carcinoma (S2). Surgical biopsies included liver metastasis (S3M), well-differentiated gastric adenocarcinoma (S3G) and a histopathologically normal celiac lymph node (S4L). TNM classification was T4N2M1. Despite early-onset, tumor histology did not support a case of Hereditary Diffuse Gastric Cancer (HDGC). Immunohistochemistry showed E-cadherin membrane expression in tumor cells. No alterations were found in MLH1, MSH2, PMS2 and MSH6 expression, ruling against an extra-colonic manifestation of Lynch Syndrome (data not shown). Figure 1 shows immunostaining for p53 in metaplasia (Figure 1A) and in gastric adenocarcinoma (Figure 1B). In the latter, all cancer cells showed strong positivity in the nucleus as well as weak cytoplasmic staining. In metaplasia, p53 was detected in the nucleus of cells in small, defined areas with intestinal differentiation (as shown by the presence of goblet cells) whereas areas of non-intestinal metaplasia were negative for p53 expression. Sequencing of *TP53 *exons 2 to 11 identified wild-type sequence (CGC) in S1 (metaplasia) while S2, S3G and S3M showed a mutation at codon 337 (c.1010G > A, p.R337H) (Figure 2A). The celiac lymph node (S4L) was negative for p.R337H (data not shown).

Presence of the founder haplotype associated with p.R337H was assessed by analysis of SNP 28 (rs9894946) in the *TP53 *gene with the T allele indicating the founder haplotype [11]. This allele was detected in the patient's samples containing p.R337H (S2, S3G and S3M) but not in those with wild-type sequence (Figure 2B). Allele-specific PCR demonstrated that p.R337H was located on the haplotype carrying SNP28, identical to the founder haplotype (data not shown). Presence of p.R337H was confirmed by Restriction Length Fragment Polymorphism (RFLP) using *HhaI*, which cuts within codon 337, in S2, S3G and S3M but not in S1 or S4L (data not shown). Genomic DNA was obtained from peripheral blood of the father, mother and of one brother. The mutant haplotype was constitutive (in heterozygosity) in the father's genomic DNA (Figure 2), whereas mother and brother were wild-type for *TP53*. DNA from peripheral blood cells of the patient was not available.

### Discussion

This case is one of the earliest cases of gastric cancer ever reported. Gastric adenocarcinoma is extremely uncommon under 21 years of age [12]. Data from Brazilian cancer registries do not show any evidence of occurrence of gastric adenocarcinoma in children or teenagers [13]. This patient was the youngest case of gastric cancer over a period of 18 years in the pathology archives of Hospital AC Camargo, one of the largest cancer centers in Brazil. The patient did not present any evidence of familial history of cancer and had no report of personal history of other disease including cancer. In previous studies, we observed that the *TP53 *p.R337H mutation is present in the germline of about 1:300 individuals (0.3%) in Southern Brazil as the consequence of a founder effect predisposing to early cancer [8]. However, the penetrance of this mutation is incomplete. In families with clinical definitions of LFS/LFL, about 25 to 30% of p.R337H carriers remain apparently cancer-free over their lifetime (compared to a penetrance of over 90% in carriers of germline *TP53 *mutations occurring at "hotspot" codons). Furthermore, the penetrance at age 30 is about 15%, compared to 50% in carriers of mutations at "hotspot" codons. Because of this incomplete penetrance, a germline p.R337H mutation may be present in individuals without specific familial cancer history, in particular in the absence of extensive pedigree data. These considerations led us to test for the presence of p.R337H in retrospective, pathology archived tissues of this very young case.

The *TP53 *mutation p.R337H, borne by a haplotype carrying the T allele of rs9894946 (SNP28) was detected in tumor and metastasis but not in non-cancer tissues of this 12-year-old patient. This haplotype corresponds to the documented founder allele detected in many Brazilian LFS/LFL families and is present in only 2% of Brazilians. On the other hand, p.R337H has never been documented as a somatic mutation in any cancer at the IARC *TP53 *database (http://www-p53.iarc.fr). Therefore, the probability that the mutation may arise as somatic mutation on the allele carrying SNP28 independently of the founder mutation is extremely low. Moreover, neither the p.R337H mutation nor the T allele of rs9804946 was detected in non-tumor tissues of the patient, although both variants were detected in cancer tissues. The presence of the founder haplotype in tumor, but not in non-tumor tissue of the patient is therefore highly puzzling. The analysis was repeated multiple times by different technicians and using different DNA extracts, ruling out a technical artifact. On the other hand, the hypothesis of a selective loss of heterozygosity for p.R337H both in metaplasia and in the non-involved celiac lymph node seems extremely unlikely. Consistent with a germline origin, the mutant haplotype was found in the father's DNA, but not in the mother and a brother. This observation indicates that the mutant haplotype detected in the patient's tumor is of paternal origin.

The mechanisms that have led to the presence of this mutant haplotype only in cancer cells of the patients are obscure. The results are consistent with the hypothesis that the patient may have harbored two populations of cells, one containing the paternal *TP53 *wild-type allele, and another one containing the paternal *TP53 *mutant allele, the latter present at very low levels, undetectable by sequencing in most tissues. The maternal, wild-type allele appears to be identical in both cell populations. This situation would be consistent with genetic microchimerism. Microchimerism refers to harboring a small number of cells or DNA from a genetically different individual, often due to maternal-fetal or feto-fetal trafficking during pregnancy [14-16]. In this patient, we suggest that microchimerism may have been caused by feto-fetal cell trafficking between dizygotic twin fetuses, one of whom had the paternal mutant haplotype and underwent very early developmental failure. It should be noted that the loss of a dizygotic fetus has not been documented in the mother's history of pregnancy, making this hypothesis unverifiable.

## Conclusions

This is, to our knowledge, the first report suggesting microchimerism for germline mutation that has led to cancer development. In contexts other than Southern Brazil, where the unique p.R337H founder haplotype is frequent and well characterized, such an event would have been undetected and interpreted as a somatic mutation. Our results therefore call for caution when assessing the genetic status of early or uncommon cancers in populations with common founder haplotypes. Testing tumor DNA in addition to genomic DNA should be considered in such patients to rule out the possibility of microchimerism.

## Consent

Written informed consent could not be obtained from the patient for publication of this case report and any accompanying images because the patient has died. The patient's mother has consented to publishing the data.

## Competing interests

The authors declare that they have no competing interests.

## Authors' contributions

EMS, MIWA, PH and FAS participated in the design of the study, analyzed the data and drafted the manuscript. Additionally EMS and GMP carried out the molecular genetic studies. MIWA was the responsible for the diagnosis, genetic counseling and management of the patient's family. ALM, MO and PAP participated in revising the manuscript critically. PH coordinated the study and drafted the manuscript. FAS advised on all aspects of the work. All authors read and approved the final manuscript.

## Pre-publication history

The pre-publication history for this paper can be accessed here:

http://www.biomedcentral.com/1471-2407/11/449/prepub
